# Reversed spin of a ratchet motor on a vibrating water bed

**DOI:** 10.1038/s41598-022-18423-1

**Published:** 2022-08-19

**Authors:** Miku Hatatani, Yasunao Okamoto, Daigo Yamamoto, Akihisa Shioi

**Affiliations:** 1 Department of Chemical Engineering and Materials Science, Doshisha University, 1-3 Tatara Miyakodani, Kyotanabe, Kyoto 610-0321 Japan; 2 Research Center for Membrane and Film Technology, Kobe University, 1-1 Rokkodai-cho, Nada, Kobe, Hyogo 657-8501 Japan

**Keywords:** Nonlinear phenomena, Chemical physics

## Abstract

A ratchet gear on a vibrating water bed exhibits a one-way spin. However, the spinning direction is opposite to that of the gear placed on the granular bed. The one-way spin is caused by the surface waves of water. Surface deformation causes transportation of the water element to rotate the gear. The spatial symmetry of the surface wave and gear geometry regulates the rotational torque. In this study, the same ratchet shows reversed motion between the granular and water beds, and the direction is not determined only by the ratchet geometry. The self-organization of the fluid medium caused by small agitation induces a nontrivial inversion of the spinning direction.

## Introduction

Spontaneous regular motion in a uniform potential field has attracted attention from the viewpoint of biological motion, where the rectification of random motions by chemical reactions is realized in apparently uniform potential fields^1–7^. For a molecular motor, a typical biological motion, a ratchet-type potential, is often assumed to be a crucial part of rectification, where the periodic change in the shape of the sawtooth potential carries the molecule. The transport direction was determined solely by the asymmetric shape of the ratchet. According to a widely accepted mechanism, the chemical reaction causes a periodic change in the ratchet shape, which determines the direction of movement with the help of thermal agitation.

The understanding of biological motion has inspired a large number of studies on active matter^8–11^. They move spontaneously through chemical reactions and physical stimuli (e.g., light irradiation). Recently, ratchet motors driven by biological and/or mechanical agitation have been studied^12–21^. A ratchet motor in a bacteria-containing solution exhibits a one-way spin^13^. Here, the moving bacteria collided with the ratchet gear to move. When the motion is only Brownian (dying bacteria), the ratchet does not exhibit one-way spin because of the limitation described by the second law of thermodynamics. Therefore, this result demonstrates the critical difference between equilibrium and non-equilibrium random motions. Recently, the authors’ group demonstrated a gear spinning on a granular bed on a vibrating disk^22^. In both cases, solid particles (bacteria and granules) collided with the side wall of the ratchet to cause a spin. For both systems, a one-way spin does not appear for a gear with a symmetric shape. The spinning direction is primarily determined by the asymmetric shape of the gear. Thus, whenever the same type of gear is used, the spinning direction is the same in both cases. Generally, particles randomly collide with the ratchet, and collisions that produce a larger rotational torque determine the spinning direction. Regardless of whether the colliding particles are living organisms or activated inorganic matter, a common mechanism seems to exist. Thus, in most cases, the spinning direction is determined by the asymmetric shape of the gears.

However, the direction of motion of a ratchet motor is not always determined by the ratchet shape alone. Theoretical studies on the reverse motion have been reported, and reversible motion with a ratchet substrate has been studied as a hot topic^23,24^, for example, the Magnus ratchet^25,26^. In this study, the same type of gear as that used for the vibrating granular bed was placed on a vertically vibrated water bed. The gear exhibited one-way spin within a restricted range of vibration frequencies and gear diameters. Surprisingly, the spinning direction was opposite to that when placed on a granular bed or in a living bacterial medium. In this study, we show the difference in agitation media between discrete and continuum matters.

The remainder of this paper is organized as follows. After a brief explanation of the experimental method, the result for the angular velocity is presented and the dependency mainly on the vibration frequency and gear diameter is discussed. In particular, the critical frequency required for the gear rotation is presented. Next, the vertical displacements of the gear and adjacent water surface are presented, and their mutual relationships are discussed. This relationship reveals the mechanism of gear spinning and its dependency on the vibration frequency. We demonstrate that the gear can rotate when the gear diameter exceeds the wavelength of the water surface. In the last section, the effect of the surface flow is discussed. Numerous surface circulations that appear random develop on a vibrating water bed. The measurement of the flow velocity and flow pattern demonstrates that these circulations are not associated with gear spin.

## Results

### Gear rotation

**Figure 1:**
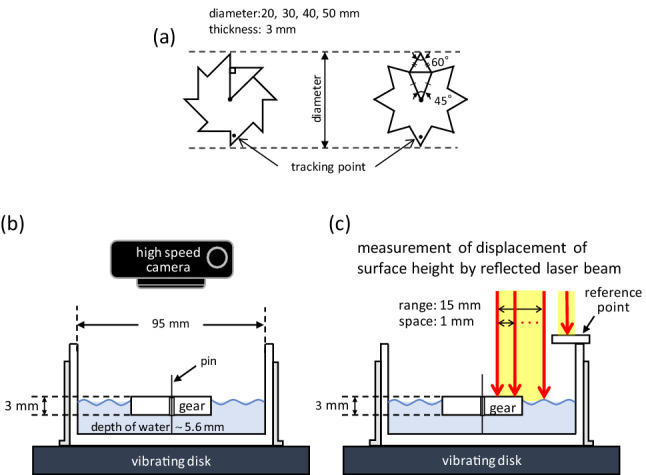
Experimental setup. (**a**) Asymmetric and symmetric gears; (**b**, **c**) experiment for video recording (**b**) and measurement of surface height profile using laser displacement meter (**c**).

**Figure 2:**
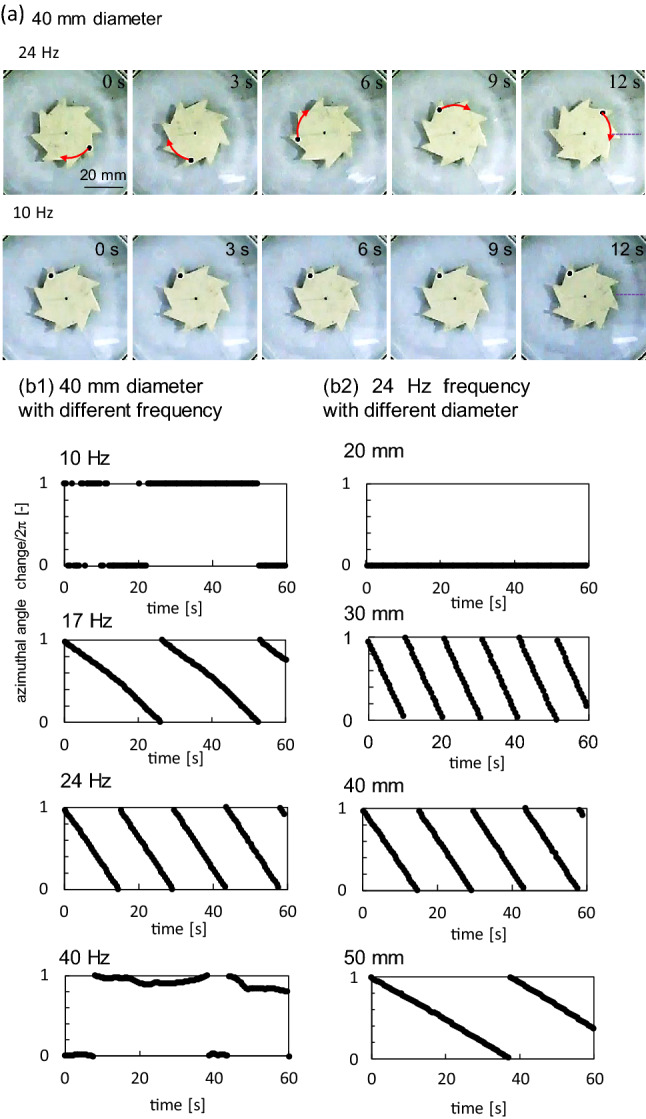
Results for the gear rotation. (**a**) Snapshot of the spinning gear with 40 mm diameter at 24 Hz (top) and fluctuating gear at 10 Hz (bottom), contrast enhanced. (**b**) Azimuthal angle (divided by 2π) of the black dot shown in (**a**). (**b1**) 40 mm-gear at various frequencies. (**b2**) Various gear diameters at 24 Hz.

The shape of the ratchet gear used in this study (Fig. 1a) is the same as that used in studies of bacterial ratchet motors and vibrating granular bed^13,22^. When a ratchet gear with an appropriate diameter is placed on water with vertical vibration (Fig. 1b), it exhibits a one-way spin under a certain vibration frequency. Figure 2a shows snapshots of the gear. When the vibration frequency (*f*_e_) is 24 Hz, a clockwise spin was observed. However, at *f*_e_ = 10 Hz, the rotation exhibits only a small fluctuation. The clockwise spin is a unique result. When living bacteria and agitated granular matter collide with a gear of the same shape, the gear exhibits anti-clockwise spin in most cases. Collision against the shorter gear edge generates a larger rotational torque than that against the longer edge. Therefore, an anti-clockwise spin can be easily expected as long as randomly moving objects hit the side face of the gear. However, in this study, the spin direction was always clockwise. The spin was not observed for the symmetric gear, as shown in Fig. 1a (Supplementary movie 1 for both types of gears).

The azimuth of the black dot in Fig. 2a (divided by 2π) is shown in Fig. 2b. The slope of the line represents the angular velocity. At a constant gear diameter (40 mm), a one-way spin was observed at 17 and 24 Hz, but not at 10 and 40 Hz. Whenever a one-way spin is observed, the angular velocity is almost constant. The angular velocity at 24 Hz is higher than that at 17 Hz. At a constant frequency of 24 Hz, the gear with a 20 mm diameter does not experience the one-way spin. For the other gears, the angular velocity decreased with an increase in gear diameter.

**Figure 3:**
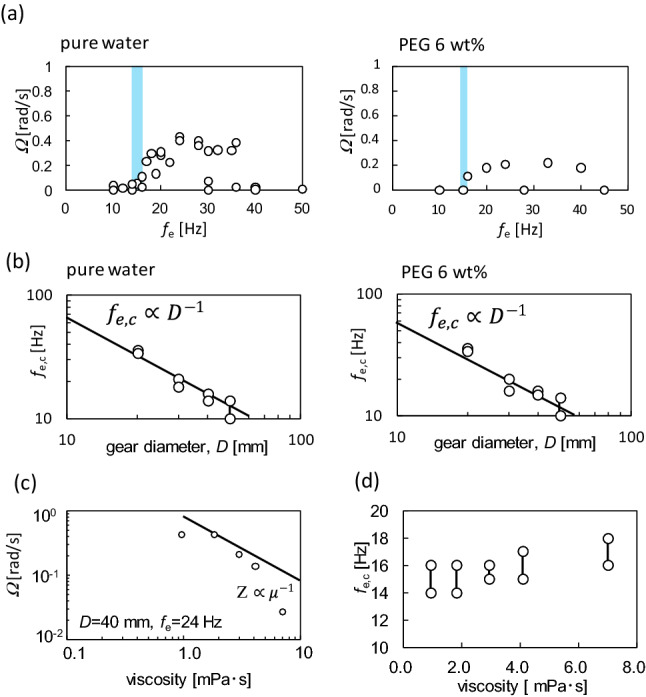
Properties of angular velocity. (**a**) Dependency of angular velocity on the frequency for pure water and aqueous solution containing 6 wt% PEG. The critical frequency is shown using the blue belt. The gear diameter is 40 mm. (**b**) Dependency of the critical frequency on the gear diameter for pure water and aqueous solution containing 6 wt% PEG. (**c**, **d**) Effects of viscosity on the angular velocity (**c**) and critical frequency (**d**), for the gear with 40 mm diameter. The PEG concentrations are 0 (the lowest viscosity), 3, 6, 8, and 12 wt%. The frequency is 24 Hz.

Figure 3a shows the angular velocity *Ω* as a function of the vibration frequency *f*_e_, where the gear diameter *D* is 40 mm (Data for other diameters are shown in SI-1). The experimental results with pure water and 6 wt% of polyethylene glycol (PEG)-containing water are shown in Fig. 3a. PEG is used mainly to increase the viscosity of water. The angular velocity increases steeply beyond a certain critical frequency *f*_e,c_. A similar characteristic in angular velocity was observed in vibrating granular beds^21,22^. This suggests that the spinning ratchet is not a result of the simple asymmetry of the gear: If the random agitation could push the gear, it would rotate toward the direction with the larger torque and/or the less dissipation, depending on the ratchet geometry. In this case, even if the agitation is small, the slow motion of the gear should be observed. However, the angular velocity of the ratchet gear starts to increase at the critical frequency in common^21,22^. This demonstrates that the ratchet gear motion is a result of dynamical pattern formation, although the pattern formation mechanism is dependent on the individual system. In this study, owing to the scattering of data, the critical frequency *f*_e,c_ was estimated within a range, as shown by the blue belt in Fig. 3. The width of this blue belt corresponds to the error range in *f*_e,c_. The *f*_e,c_ values for all gear diameters are shown in Fig. SI-1. Figure 3b shows *f*_e,c_ (with the range) as a function of the gear diameter (*D*). The result shows that *f*_e,c_ decreases monotonically with an increase in the diameter: the data are correlated by the line of *f*_e,c_ ∝ *D*^−1^. Figures 3c and d show the effect of viscosity (*μ*) on angular velocity and *f*_e,c_. The angular velocity decreases more steeply than the line $\Omega \propto {\mu }^{-1}$ at *μ* ≳ 2 mPa s. In contrast, *f*_e,c_ is almost constant except the result at the extremely high viscosity.

As shown in Fig. 3a, the angular velocity was scattered, even at the same frequency. However, this did not affect the range estimated for *f*_e,c_. We observed gear rotation every 2 Hz at a fixed gear diameter. The experiments were performed five times to determine *f*_e,c_, which was the lowest frequency with gear spinning. All the values were within the error range. Image analysis to obtain the time course of the angular velocity took a long time; hence, the data shown in Fig. 3a and SI-1 are restricted. However, we consider the range of *f*_e,c_ to be reliable. Although the angular velocities are rather scattered, as shown in Fig. 3a and SI-1, their absolute values are not discussed in this paper. Only the critical frequency is focused upon in the following discussion.

### Oscillations of gear and water surface

The behavior of a liquid drop and small solid particles (powder) on a vibrating water bed was studied^27–35^. For a bulky solid object, the dynamics of the vibrating disk have been studied well^36–42^. However, to the best of our knowledge, the behavior of bulky solid objects on vibrating water beds has been less studied.

**Figure 4:**
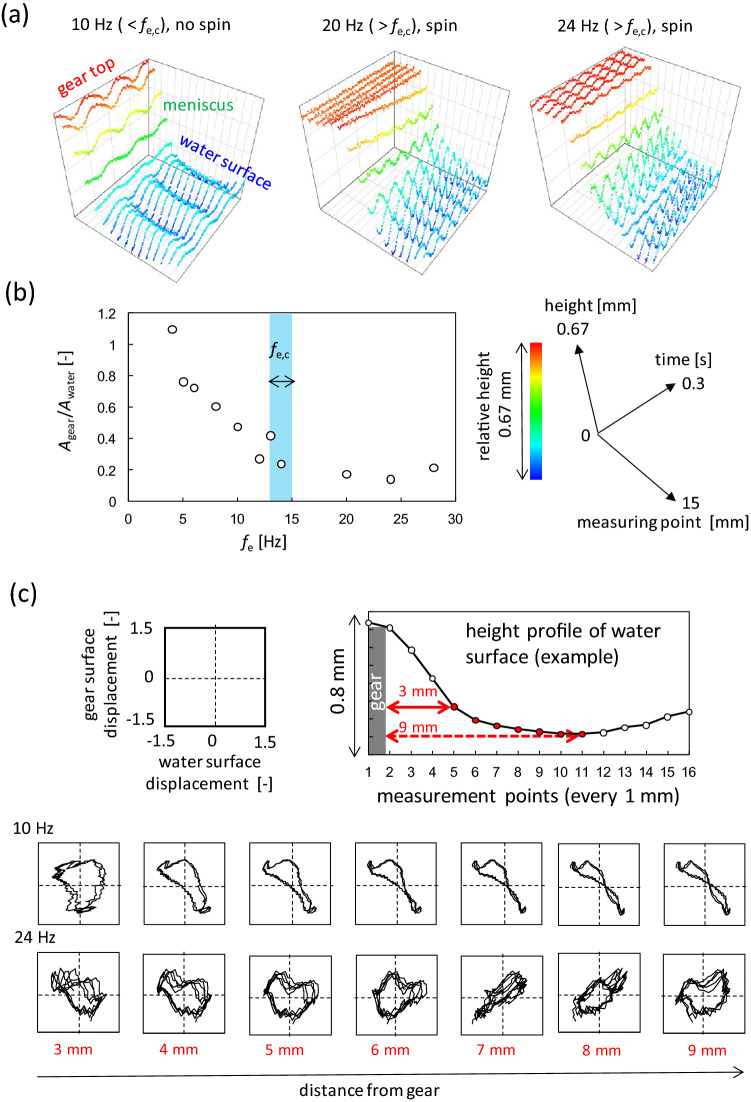
Height profile of water and gear surfaces measured by the laser displacement meter. (**a**) 3D graphics. The coordination system and their scales are common (shown on the right). The height is shown along the color bar. The gear diameter is 40 mm, and *f*_e,c_ is approximately 15 Hz. (**b**) The ratio of maximum vibration displacements, *A*_gear_/*A*_water_, is plotted against *f*_e_. The blue belt indicates *f*_e,c_ determined from Fig. 3a. (**c**) Lissajous figure for the oscillations between the gear and the water surface. The coordinate system and scale are common. The measurement positions are shown at the top.

Figure 4a shows the time course of the height profile of the line that connects the points on the gear and water surfaces. This line is shown in Fig. 2a as a purple dotted line. The height profile was measured using a laser-displacement meter. The schematic of this method is shown in Fig. 1c (the duration for obtaining the data is 0.3 s. Thus, the position of the gear edge may be regarded as the same even when the gear rotates.) When *f*_e_ < *f*_e,c_, the gear and water surfaces oscillated vertically. Both oscillations are in the antiphase and form stationary oscillations. This anti-phase oscillation is shown in the Lissajous figure in Fig. 4c (10 Hz). This figure represents the phase relationship between the oscillations of the gear and water surface. The graph becomes diagonal, anti-diagonal, and circular for the phase differences 0, π, and π/2, respectively (the method to draw the Lissajous figure is explained in SI-2). The oscillation of the water surface was measured from 3 to 9 mm from the gear edge, as indicated in Fig. 4c (the duration for obtaining the data for the Lissajous figure was 0.2 s. Thus, the position of the gear edge may be regarded as the same even when the gear rotates). The 10 Hz Lissajous figures exhibit a clear anti-phase oscillation between the gear and water surface.

By contrast, at *f*_e_ = 20 Hz (> *f*_e,c_) in Fig. 4a, the gear height is almost constant. The gear did not oscillate significantly against the water surface, although oscillations with small amplitudes were observed. The anti-phase relationship shown at 10 Hz was not observed. The same trend is observed at 24 Hz, as shown in Fig. 4a and c where the Lissajous figure does not show a regular pattern. Therefore, an anti-phase relationship was observed only at *f*_e_ < *f*_e,c_. This trend was confirmed in almost all experiments performed in this study. This indicates that the critical frequency *f*_e,c_ is the maximum value for the gear to oscillate in the antiphase against the water surface.

The maximum vibration displacement of the gear (*A*_gear_) is shown in Fig. 4b as a ratio to that of the water surface (*A*_water_). *A*_gear_/*A*_water_ monotonically decreased with an increase in *f*_e_ to reach a constant value at *f*_e_ ~ *f*_e,c_. The low value at *f*_e_ > *f*_e,c_ indicates that the gear does not oscillate significantly against the water surface. When *A*_gear_/*A*_water_ reaches this value (~ 0.2), the anti-phase oscillation of the gear is lost. In other words, the anti-phase oscillation and the larger oscillation amplitude of the gear are coupled.

**Figure 5:**
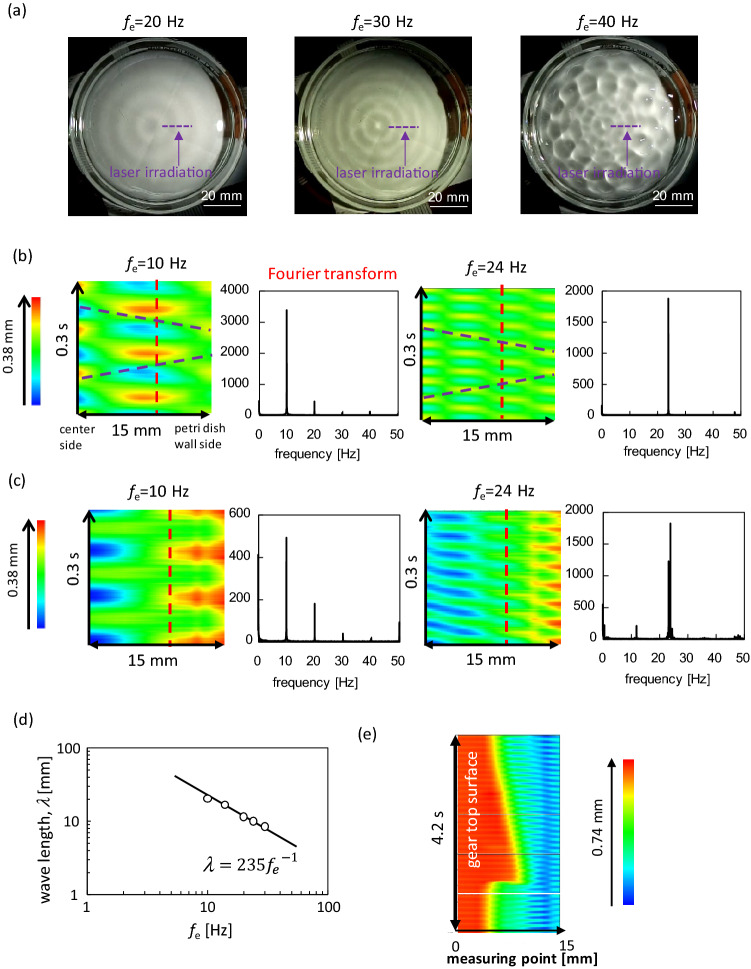
Pattern formation on the water surface. (**a**) Snapshots of the surface pattern. The line for laser irradiation for the displacement meter is shown (without gear). (**b**) Space–time plot at the irradiation line and the Fourier spectrum at the red dashed line without gear. (**c**) Those with the gear. The laser irradiation line was between 2 mm apart from the gear edge and 10 mm from the sidewall of Petri dish. (**d**) Relationship between wavelength and frequency in the absence of the gear. (**e**) Space–time plot on the line involving the gear. The red portion is on the gear. The space–time plots with the gear is affected by the meniscus shape formed by the gear. Hence, detecting the wave form by visual inspection is difficult compared with the gear-free case.

Surface waves generated by vertical vibrations exhibit various types of patterns on the water surface^29,43–48^. When the oscillation frequency of the water surface (*f*_w_) is half of the external frequency (*f*_e_/2), it is called a Faraday wave. The wave pattern, which depends on the vibration frequency, has been extensively studied. In this experiment, the relationship between *f*_w_ and *f*_e_ is determined. The results reveal that *f*_w_ = *f*_e_/2 for *f*_e_ > 30 Hz and *f*_w_ = *f*_e_ for *f*_e_ < 30 Hz (SI-3). Figure 5a shows a photograph of the water surface. When *f*_e_ < 30 Hz (*f*_w_ = *f*_e_), the pattern is circular. However, when *f*_e_ ≳ 30 Hz (*f*_w_ = *f*_e_/2), the circular texture was violated and restricted to the central portion (Supplementary movie 2). A one-way spin is obtained with almost perfect reproducibility when the circular pattern dominates the entire water surface. This circular pattern was not violated by the presence of the gear.

Figures 5b shows the space–time plot of the height of the water surface without the gear. The measurement line is shown in Fig. 5a as a laser irradiation line. When *f*_e_ = 10 Hz (< *f*_e,c_) and 24 Hz (> *f*_e,c_), circular patterns were formed, and the space–time plot along the radial direction was independent of the azimuth. The space–time plot is that of a simple stationary wave for both frequencies. A stationary wave is formed by two oppositely traveling waves of the same wavelength and frequency. The wave velocity of each wave may be estimated from the line diagonally connecting the wave crest (or bottom) in the space–time plot. More precisely, the velocity was obtained from the relationship between the wavelength and frequency, as shown in Fig. 5d. Here, the wavelength *λ* is estimated from the space–time plot, and the frequency is *f*_e_. The oscillation frequency of the water surface *f*_w_ is equal to *f*_e_ when *f*_e_ ≲ 30 Hz. This is shown in the Fourier spectra in Fig. 5b. The result in Fig. 5d is well correlated by the line *λ* (mm) = 235/*f*_e_ (Hz). This indicates that the wave velocity was 235 mm/s. The diagonal line that expresses this velocity, which well connects the wave crests (or bottoms), is shown in Fig. 5b. This value agrees with the theoretical calculation for the surface wave of shallow water ($\sqrt {gH} = \sqrt {9800{\text{ mm}}/{\text{s}}^{2} \cdot 5.6 {\text{mm}}} = 234\frac{{{\text{mm}}}}{{\text{s}}}.{\text{ Here}},{ }H{\text{ is the depth of water}}$).

Figure 5c shows the space–time plot of the height of the water surface with the gear. The measurement line was located on the water surface, which did not contain the gear. When *f*_e_ = 10 Hz, the one-way spin was not observed. The pattern exhibits a simple stationary wave. The space–time plots with the gear are affected by the meniscus shape formed by the gear; hence, the pattern is not simple, even when a stationary wave is formed. However, the space–time plot is that of a stationary wave because almost the same horizontal (spatial) color profile is repeated periodically in time. A one-way spin was observed at 24 Hz and the space–time plot was then slightly distorted. However, the basic feature of the stationary wave pattern was maintained, that is, the positions with the extrema of the oscillation were almost unchanged. This distortion is probably caused by the effects of the spinning gear. Figure 5e shows the space–time plot measured along a line, including the gear surface. As the gear rotates, the gear region in Fig. 5e advances and recedes, tracing the gear shape. Distortion of the space–time plot was also observed. The inclination of the same colored part is opposite at the tip and at the dent of the gear. As shown later, surface flow caused by the spinning gear was observed. The flow directions were opposite at the tip and dent of the gear. This surface flow, which is shown later, distorts the space–time plot. Although a small distortion was observed, the space–time plot can be understood as a stationary wave pattern.

**Figure 6:**
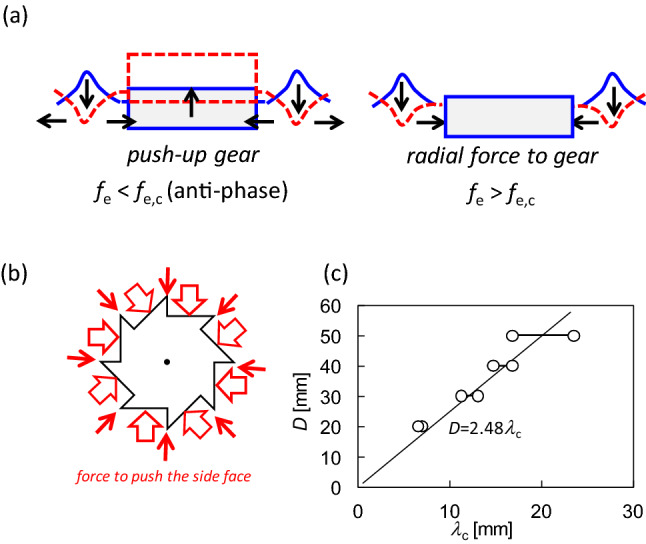
Schematic representation of the mechanism of the spinning gear and the related result. (**a**) Schematic representation of the effect of damping wave crest on the gear motion. (**b**) Pressure acting on the side face of the gear. (**c**) Relationship between the gear diameter and wavelength at the critical frequency of the diameter.

Considering the volume conservation of water, the upward and downward motions of the water surface accompany the radial motion when the surface wave has a circular pattern, as shown in Fig. 5a (*f*_e_ ≲ 30 Hz). Such fluid motion is often modeled by the trochoid motion of a fluid element, that is, circular motion in the vertical plane^49^. Even if the wave is not a typical trochoid, volume conservation must cause horizontal transport of the water volume. The horizontal motion oscillates when a stationary wave is formed. Figure 6a (left) shows the antiphase oscillation. Considering a concentric ring-wave pattern, the damping wave crest excludes the water volume under the crest toward the radial direction. Nearly half of the water volume moved toward the gear. If the gear moves upward, water volume enters under the gear. This results in the anti-phase oscillation between the gear and water surface. Therefore, the anti-phase oscillation requires a larger gear amplitude to absorb the water volume excluded by the damping wave. When the vibration amplitude of the gear is small compared to the amplitude of the water surface, the water volume does not enter under the gear. This is shown in Fig. 6a (right), where the water volume pushed the side face of the gear. As shown in Fig. 6b, the shorter edge of the gear was not pushed effectively because it was parallel to the radial direction. However, this pressure effectively pushes the longer edge. This results in clockwise rotation of the gear.

When the gear diameter is smaller than or comparable to the wavelength of the surface wave (*λ*), the gear rides on the surface wave. This results in anti-phase oscillation of the gear against the water surface. Therefore, to push the side face of the gear, the gear diameter should be sufficiently larger than the wavelength; that is, a one-way spin appears when the ratio *D*/*λ* is much larger than unity. As the wavelength becomes shorter with an increase in frequency, the critical frequency *f*_e,c_ exists at a given diameter *D*, that is, beyond *f*_e,c_, *D*/*λ* can take values much larger than unity. Figure 6c shows the relationship between *D* and *λ*_c_, where *λ*_c_ is the wavelength at *f*_e_ = *f*_e,c_ and is calculated as *λ*_c_ (mm) = 235/*f*_e,c_ (Hz). The maximum and minimum wavelengths were plotted as the critical frequencies for a range. All the plots can be correlated using a unique line, *D* = 2.5 *λ*_c_. The gear does not ride on the surface wave when *D* ≳ 2.5*λ*_c_. Then, the radial motion of the water volume cannot enter under the gear and push the side-face.

The addition of PEG violated the circular pattern on the water surface, which was observed at *f*_e_≲30 Hz (Fig. 5a). Photographs and space–time plots for the PEG-containing experiments are shown in SI-4. An almost perfect circular pattern was observed only at *f*_e_ < 15 Hz. This may have been caused by the decrease in the surface tension by PEG, which was supported by the increase in the oscillation amplitude of the water surface at higher frequencies. Such a violation of the circular pattern reduces the angular velocity because the water volume excluded from the damping wave crest can move in the peripheral direction in addition to radial motion. The angular velocity then decreases more steeply than the line $\propto \mu^{ - 1}$, as shown in Fig. 3c. Owing to the violation of the pattern, it is difficult to estimate the wavelength over a wide range of frequencies from the space–time plot on a radial line. Thus, the space–time plot for the lower frequency is shown in SI-4, where the circular pattern is maintained. The wavelength was almost the same as that of the PEG-free water at the same frequency. This result suggests that *f*_e,c_ in PEG-free and PEG-containing water is nearly identical because *f*_e,c_ is determined by the relationship between *D* and *λ* (Fig. 6c). This expectation is supported by the results shown in Fig. 3d, which show that the effect of PEG addition on *f*_e,c_ is small (almost none).

The pressure that pushes the side face rotates the gear. However, the rising wave crest pulled the water volume from the gear. The experimental results indicate that the pushing pressure effectively rotates the gear, whereas the pulling force does not work well. When water pushes the gear, the volume of water directly collides with the side face. For the back flow, however, the compensated flows originate from anywhere around the gear: from a deeper place and from under the gear. In this case, it may be difficult for the back flow to generate an effective pulling force, even if there is adhesion between the water and the gear.

### Surface flow by vibration

**Figure 7:**
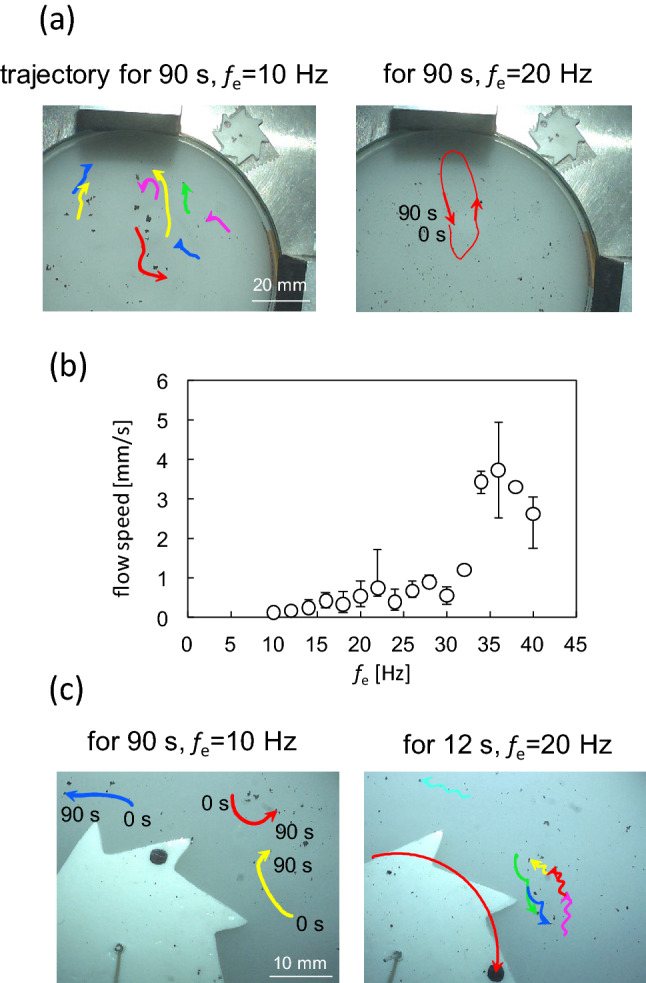
Motion of charcoal (tracer) particles on the water surface. (**a**) Trajectories in the absence of the gear at 10 Hz (left) and 20 Hz (right). (**b**) Mean speed with the error bar for the tracer particles as a function of the vibration frequency in the absence of the gear. (**c**) Trajectories of the tracer particles in the presence of the gear at 10 Hz (left) and 20 Hz (right).

It has been reported that vortices (circulation) spontaneously form on the surface of a vibrating water bed^27–29,50,51^. Subsequently, numerous randomly distributed circulations were developed. In this section, the effect of the surface flow on the one-way spin is discussed. Circulation was also observed in this study. The trajectories of the tracers, which are shown in Fig. 7a, appear randomly distributed without the gear. (Supplementary movie 3) Fig. 7b shows the mean speed of the flow against the vibration frequency *f*_e_: more than two tracer particles were selected in a movie. The tracers were tracked for 14 s (fastest particle) to 90 s (slowest particle). The velocities were calculated every 0.5 s by image analysis. The maximum and minimum velocities are shown as error bars in Fig. 7b. The mean speed gradually increased at low frequencies and exhibited an almost discontinuous change at approximately *f*_e_ = 30 Hz. This probably results from Faraday wave formation. However, the angular velocity of the gear did not exhibit such a discontinuous change at approximately *f*_e_ = 30 Hz (Fig. 3a). Furthermore, for the critical frequency (*f*_e,c_) at which the angular velocity of the gear starts to increase, there is no significant change in the speed of circulation around *f*_e_ = *f*_e,c_ (*f*_e,c_ depends on the gear diameter, and they are distributed from 10 to 40 Hz, as shown in Fig. 3b). In addition, the mean speed was lower than the speed of the gear tip. This is estimated by the angular velocity multiplied by the gear radius, approximately 8 mm/s, as shown in Fig. 3a. Circulation formed on the water surface is not a direct power source for the spinning gear.

Figure 7c shows the trajectories of the tracer particles with the gear (refer to Supplementary movie 4). In the absence of one-way spin (*f*_e_ = 10 Hz), the pattern is similar to that without the gear. For the one-way spin of the gear, the trajectory exhibits a zigzag shape. The movie (Supplementary movie 4) shows the zigzag results caused by the gear shape. When the tip of the spinning gear approaches the tracer particle, it departs from the tip by an outward flow. In contrast, the tracer particles are pulled toward the dent of the gear. The distortion of the space–time plot in Fig. 5e seems to be associated with these surface flows because the inclination of the same colored part is opposite at the tip and at the dent of the gear. The surface flow may perturb the wavelength and/or frequency at a fixed point (it was confirmed with a sinusoidal wave that such a perturbation can cause the inclination of the same color part in the space–time plot). The form of the stationary wave on the water surface was likely affected by these surface flows. However, these flows are not the power source for the spin but the result of the spin. This is reasonably accepted by the observation that once the external vibration is turned on, the gear starts to rotate immediately (Supplementary movie 5). The development of such a highly regulated flow pattern within a short period is difficult.

### Results at higher frequency

The angular velocity increases monotonically at *f*_e_ > *f*_e,c_. However, the angular velocity is smaller (almost zero) at extremely high frequencies (> 40 Hz), as shown in Fig. 3a and SI-1. When *f*_e_ ≳ 30–40 Hz, a complicated wave pattern often appears instead of a circular texture on the water surface, as shown in Fig. 5a. This probably reduces the stability of the one-way spin because the radial motion of the water volume may be violated by this complicated wave pattern, that is, the water volume by the damping of the wave crest (Fig. 6a) may move toward the circumferential direction. This event destabilizes the one-way spin.

The formation of the noncircular pattern of the surface wave is probably the main reason why the one-way spin is destabilized in the high-frequency range. However, another factor may affect this result, which is based on the simple consideration that the falling gear cannot catch up to the descending water surface. Consider a disk that falls under gravity. The condition that the falling gear cannot maintain contact with the water that descends by mechanical motion is expressed by

$$ f_{max} = \frac{1}{2\pi }\sqrt{\frac{g}{a}}  $$

(SI-5), where *a* denotes the amplitude of the external vibration. Under the present experimental conditions, *f*_max_ becomes 40.7 Hz. This frequency approximately agrees with the frequency beyond which the one-way spin is destabilized.

Even when *f*_e_ > *f*_max_, the adhesion force between the gear and water may maintain contact. This may increase the actual *f*_max_. Because the precise estimation of *f*_max_ is not easy in experiments, further discussion is difficult at present.

## Discussions

Mechanical vibration causes numerous surface circulations that are generated almost randomly in water. If these flows can rotate the gear, they can cause an anti-clockwise spin. However, in the present system, the free motion of the water surface forms a circular pattern accompanying the transverse wave. The periodic up and down of the surface acted as a pump to push the water element along the radial direction. Although this water moves only for a short range, it is sufficient to rotate the gear, given the ratchet geometry. Generally, fluids have a higher potential for dissipative pattern formation (self-organization) than discrete media, such as powder. High self-organization of the fluid may realize a wide variety of regulated motions of an asymmetric object. Even when living organisms or artificial active matter are utilized as agitating elements, their collective motions help extract the regulated motion of a larger object compared to the use of the individual motion. Here, the interaction of agitating elements causes a collective motion. The present study on the ratchet gear may be based on an extension of this consideration, that is, highly interacting elements are more fascinating as the agitating medium than those with random and independent particles.

## Methods

Figure 1a shows the gears used in the experiment. The gear shape was based on that proposed by Leonardo et al.^13^. Two types of gears were used: ratchet and symmetric gears. The gear diameters were 20, 30, 40, and 50 mm. They were made of acrylonitrile butadiene styrene resin and fabricated using a 3D-printer (XYZ-Printing, 1.0 pro 3-in-1). The gear surface was polished using sandpaper to obtain a smooth surface. The gear was placed in a water-filled Petri dish with a diameter of 95 mm and depth of 30 mm, as shown in Fig. 1b and c. A hole with a diameter of approximately 1 mm was drilled at the center of the gear. The gear was placed at the center of the Petri dish using a thin metal wire that passed through the hole. The wire stood vertically in the magnetic field generated by the vibrator. The water was fully deionized (ELGA Labwater, Purelab Flex 3). PEG (MW 8000, MP Biomedicals) was dissolved in water to increase viscosity as needed. Briefly, 40 mL of water was poured into a Petri dish. The change in the height profile of the water surface and gear top was measured using a laser displacement meter (Keyence, LJ-V7080). The height profile was measured every 1 mm over a 15 mm range at a frequency of 10^3^ Hz. (Data acquisition was performed every 1 ms.) The height measured is the distance between the measured surface and reference point moving with the vibrating substrate, as shown in Fig. 1c. Thus, the time course of the height is the change in the vertical position of the surface relative to the vibrating substrate. When the laser displacement meter was used, a small amount of white water paint was mixed with water to enhance reflection. The effect of paint on gear dynamics was negligible. The surface flow was visualized using carbon powder as a tracer. The setup is fixed to a vibrating disk. Vertical vibrations were applied to the disk, as shown in Fig. 1b and c. The vibrator (513-B, EMIC Co.) comprised a function generator (DF1906, NF Co.) to generate a sinusoidal wave, and an accelerometer with a charge amplifier (505-CBP, EMIC Co.). The amplitude was controlled to be 0.15 mm using a power amplifier (371-A/G, EMIC Co.). The vibrating disk, where the Petri dish was fixed, was agitated vertically at a predetermined frequency and amplitude.

The motions of the gear and carbon powder were monitored using a digital camera (CASIO EX-100F), which can capture motion pictures, and a high-speed camera (Keyence VW-6000), respectively. The position of a fixed point at the tip of the gear and the tracer particles were traced using TEMA (Photron) and Move-Tr/2D (Library Co.) software. The azimuth of the tracking point on the gear is measured and used to calculate the angular velocity. The 3D-figures and space–time plots were obtained using the free software RINEARN. The viscosities of the water and PEG solutions were measured using a tuning-fork vibration viscometer (A&D Company, Ltd. SV-10A). All experiments were performed at room temperature.

## Supplementary Information

Supplementary Information 1.

Supplementary Video 1.

Supplementary Video 2.

Supplementary Video 3.

Supplementary Video 4.

Supplementary Video 5.

## Data Availability

The datasets used and/or analyzed during the current study are available from the corresponding author upon reasonable request.
