# Supplementary material for: Reversed spin of a ratchet motor on a vibrating water bed
Source: Sci Rep. 2022 Aug 19;12:14141. doi: 10.1038/s41598-022-18423-1 (PMC9391431; doi:10.1038/s41598-022-18423-1)

Supplementary Informtion

Reversed Spin of a Ratchet Motor on a Vibrating Water Bed

Miku Hatatani, Yasunao Okamoto, Daigo Yamamoto, and Akihisa Shioi*
Department of Chemical Engineering and Materials Science, Doshisha University
1-3 Tatara Miyakodani, Kyotanabe, Kyoto 610-0321 Japan
E-mail: ashioi@mail.doshisha.ac.jp

Supplementary Movies

Supplementary movie 1
Movie of the symmetric and ratchet gears

Supplementary movie 2
Surface pattern of water under vibration

Supplementary movie 3
Tracer motions without gear at 10 Hz and 20 Hz

Supplementary movie 4
Tracer motions with gear at 10 Hz and 20 Hz

Supplementary movie 5
Gear response to the vibration

SI-1

Angular velocity and frequency for all gear diameters

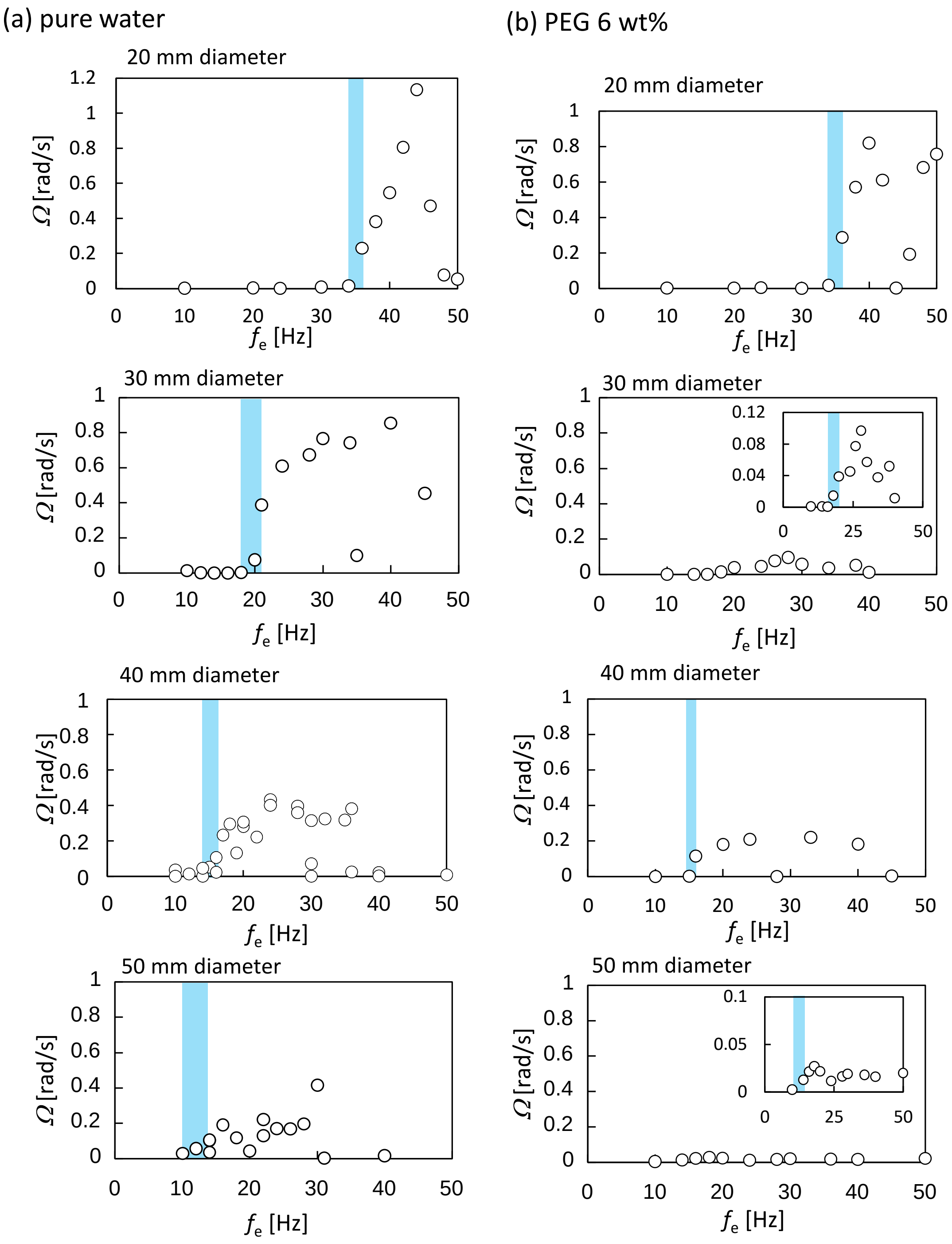

SI-2

Method to draw Lissajous's figure

The oscillations of water surface and the gear top were plotted against the time. The waves for 0.2 s at a common time window were randomly selected. In this time window, the vertical displacements of the water surface and the gear top were smoothed every 5 ms. The smoothed waves were scaled in such that the maximum and the minimum of each wave became +1 and −1, respectively. The scale of the displacement was provided in the proportional manner. The characteristic of the Lissajous figure discussed in the main text was the same independently of the selected time window.

SI-3

Frequency of water surface and gear

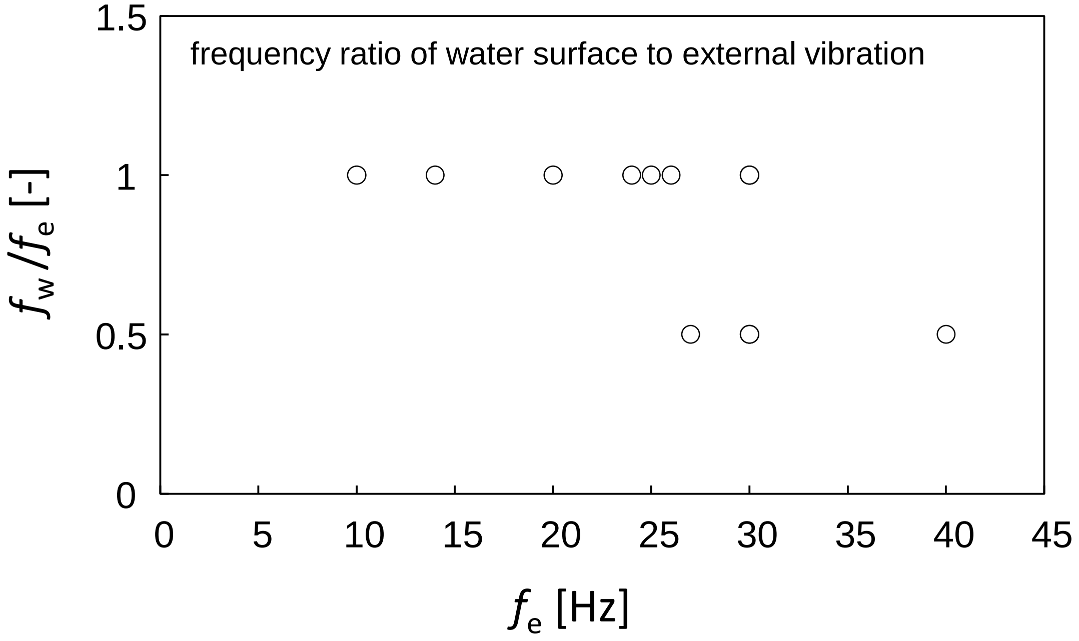

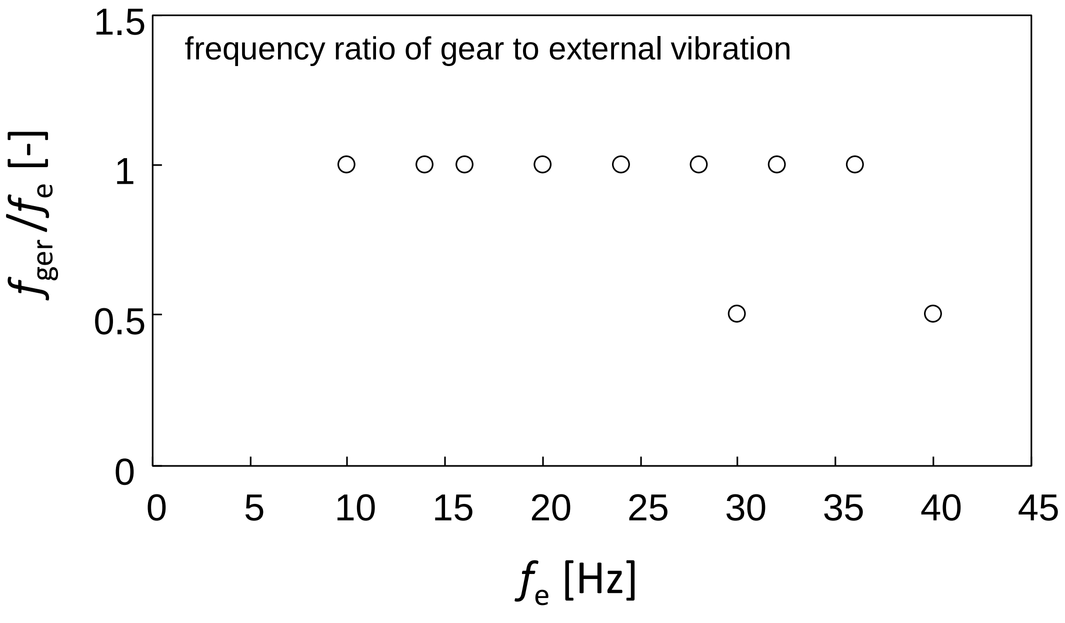

SI-4

Surface wave pattern and space–time plots for 6 wt%-PEG solution. The space–time plot of PEG-free water is shown as reference.

Photographs of surface pattern for PEG-containing solution

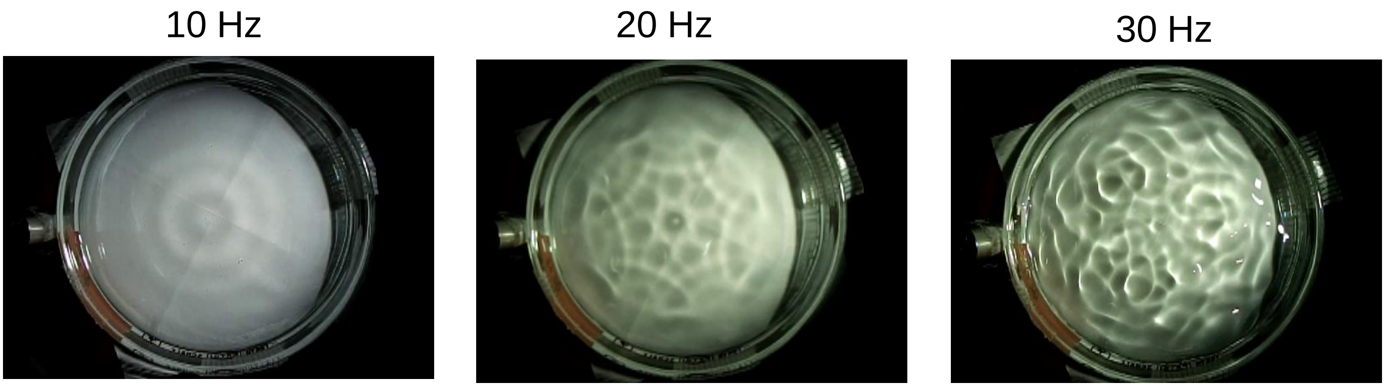

Patterns at 20 Hz and 30 Hz change with time complicatedly. These are examples.

Space-time plot at low frequency

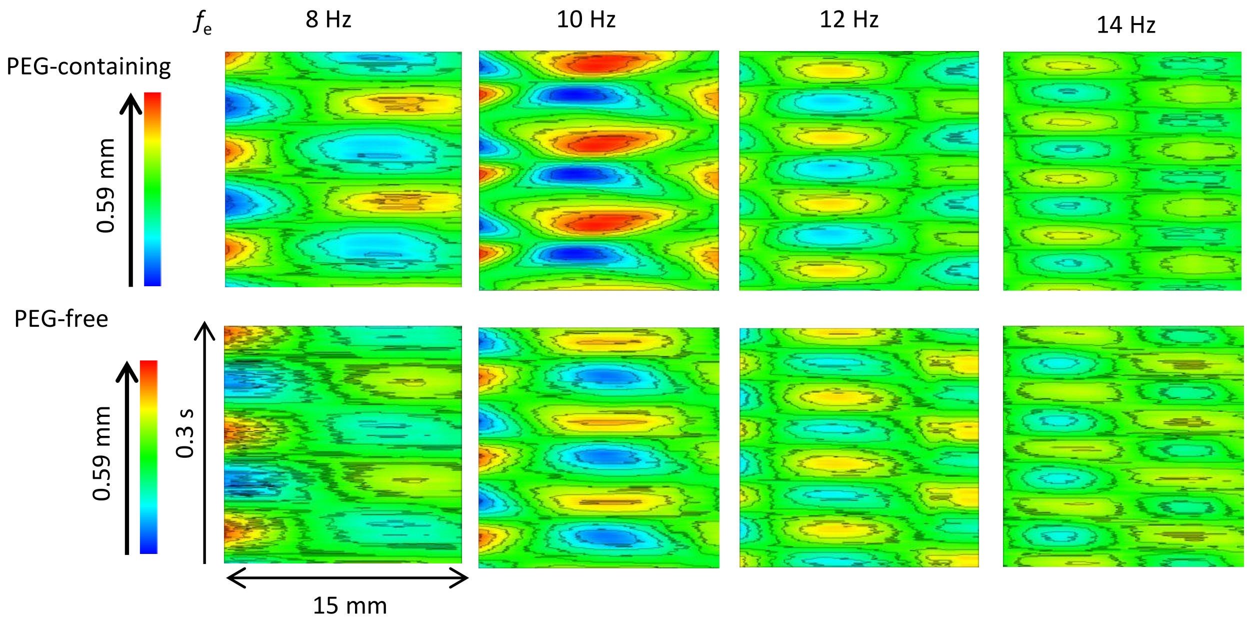

SI-5

Maximum frequency for the falling gear to keep contact with the water surface

The velocity of a falling gear is given by $v_g = -gt$. Here, t is taken as zero at the top position. As the external vibration is expressed by $a\cos(2\pi f_e t)$, the velocity of water surface under the gear is given by $v_w = -2\pi a f_e \sin(2\pi f_e t)$. When $|v_g| > |v_w|$ is always satisfied, the direct contact of gear and water is maintained. This condition is expressed by

$$\left|\left(\frac{dv_g}{dt}\right)_{t=0}\right| > \left|\left(\frac{dv_w}{dt}\right)_{t=0}\right|$$

This yield

$$g > a(2\pi f_e)^2$$

$f_{\max}$ is derived from this equation.

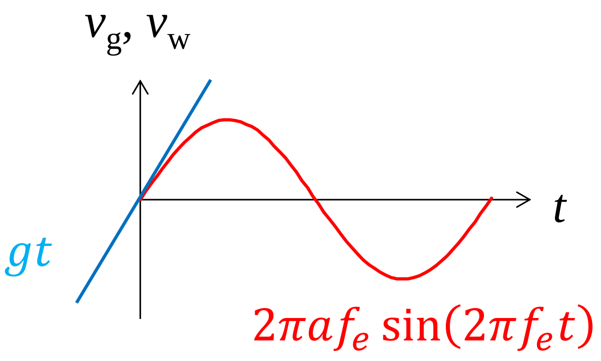

Supplement: Supplementary file 1 — Supplementary Information 1. [file 41598_2022_18423_MOESM1_ESM.pdf]
